# Extrapolating non-target risk of *Bt* crops from laboratory to field

**DOI:** 10.1098/rsbl.2009.0612

**Published:** 2009-09-09

**Authors:** Jian J. Duan, Jonathan G. Lundgren, Steve Naranjo, Michelle Marvier

**Affiliations:** 1 USDA-ARS, Beneficial Insects Introduction Research Unit, Newark, DE 19713, USA; 2 USDA-ARS, North Central Agricultural Research Laboratory, Brookings, SD 57006, USA; 3 USDA-ARS, Arid-Land Agricultural Research Center, Maricopa, AZ 85238, USA; 4 Environmental Studies Institute, Santa Clara University, Santa Clara, CA 95053, USA

**Keywords:** non-target effects, *Bt* crops, risk assessment, transgenic crops, meta-analysis

## Abstract

The tiered approach to assessing ecological risk of insect-resistant transgenic crops assumes that lower tier laboratory studies, which expose surrogate non-target organisms to high doses of insecticidal proteins, can detect harmful effects that might be manifested in the field. To test this assumption, we performed meta-analyses comparing results for non-target invertebrates exposed to *Bacillus thuringiensis* (*Bt*) Cry proteins in laboratory studies with results derived from independent field studies examining effects on the abundance of non-target invertebrates. For Lepidopteran-active Cry proteins, laboratory studies correctly predicted the reduced field abundance of non-target Lepidoptera. However, laboratory studies incorporating tri-trophic interactions of *Bt* plants, herbivores and parasitoids were better correlated with the decreased field abundance of parasitoids than were direct-exposure assays. For predators, laboratory tri-trophic studies predicted reduced abundances that were not realized in field studies and thus overestimated ecological risk. Exposure to Coleopteran-active Cry proteins did not significantly reduce the laboratory survival or field abundance of any functional group examined. Our findings support the assumption that laboratory studies of transgenic insecticidal crops show effects that are either consistent with, or more conservative than, those found in field studies, with the important caveat that laboratory studies should explore all ecologically relevant routes of exposure.

## 1. Introduction

Prior to commercialization in the USA, Canada and European Union, the potential environmental risks of transgenic insect-resistant crops are quantified via a tiered assessment (US EPA 1998, 2007; Rose 2006). A first step is the determination of the potential hazard or toxicity of the insecticidal traits (e.g. Cry proteins for crops expressing transgenes derived from *Bacillus thuringiensis* (*Bt*)) to non-target organisms (NTOs). Tier-I assessments of the NTO risk of transgenic insecticidal crops are conducted in the laboratory where surrogate NTOs representing particular taxonomic or functional guilds are subjected to insecticidal proteins or plant tissues under worst-case (typically greater than 10 times expected) exposures (Romeis *et al*. 2008). If no toxicity to NTOs is identified in Tier-I assessments, the transgenic insecticidal crop may be judged to have minimal risk. Alternatively, if potential toxicity to NTOs is identified, additional higher tier testing (e.g. semi-field or field experiments) is conducted to further characterize risk under more realistic exposures (Rose 2006). This tiered approach to risk assessment for insect-resistant transgenic crops has been advocated internationally (Romeis *et al*. 2008), but debate continues over the use of laboratory studies in predicting ecological effects in the field (Andow & Hilbeck 2004; Romeis *et al*. 2006).

We used meta-analyses to test whether laboratory studies of non-target effects of *Bt* Cry proteins are consistent with results from field studies that compare the abundance of NTOs in *Bt* crops versus non-*Bt* counterparts. Because surrogate species are often used in laboratory studies, comparisons of effects measured in laboratory and field studies often involve different species-by-toxin combinations. Our analyses test the central assumption of tiered risk assessment, which holds that laboratory assays employing surrogate species provide estimates of NTO effects that are either accurate or conservative (meaning more negative) relative to those measured in the field.

## 2. Material and methods

Data were extracted from laboratory and field studies that evaluated the effect of *Bt* Cry proteins or plant tissues containing the expressed Cry proteins. Data were drawn from the ‘*Nontarget effects of* Bt *crops database*’ (Marvier *et al*. 2007), supplemented with additional peer-reviewed field studies and laboratory studies (peer-reviewed and industry studies obtained from the US Environmental Protection Agency) reported through late 2008.

We restricted analyses to classes of proteins for which both laboratory and field data were available, which eliminated data concerning Cry9, Cry34 and Cry35. We also excluded data for varieties ‘stacked’ with other types of plant-incorporated protectants (e.g. trypsin inhibitors and vegetative insecticidal proteins). Studies included in our analyses involved Lepidopteran-active (Cry1 or Cry2 class) and Coleopteran-active (Cry3 class) proteins expressed either in *Bt* plant tissues or produced by genetically modified strains of *Bt* or *Escherichia coli*. We enforced a minimum of five observations in both the laboratory and field per functional group, thereby eliminating data for omnivores tested against Lepidopteran-active proteins, parasitoids tested against Coleopteran-active proteins and all data concerning pollinators. Studies included in our analyses presented treatment means accompanied by standard deviations (*s*) and sample sizes (*n*). When studies provided incomplete information, authors were contacted directly. We required *n*_1_ > 0, *n*_2_ > 0, *n*_1_ + *n*_2_ > 2 and *s*_1_(*n*_1_ − 1) + *s*_2_(*n*_2_ − 1) > 0. Data and additional details of study selection are reported in the electronic supplementary material.

In laboratory assays, NTOs were exposed in confined arenas to *Bt* plant tissues or diet substrates containing Cry protein and a negative (non-*Bt*) control. To maximize consistency among studies and reduce issues of non-independence, we restricted analyses to measures of survival or longevity. Laboratory assays for non-target parasitoids and predators were categorized as direct-exposure (NTOs were fed diets or plant substrates containing *Bt* Cry proteins) or tri-trophic exposure (NTOs were given access to hosts or prey that had ingested *Bt* Cry proteins) studies. Sufficient data (*n* ≥ 5) were available to compare these two study designs for Lepidopteran-active, but not Coleopteran-active proteins. Filtering data by the above criteria resulted in 74 laboratory studies (unpublished reports or published papers) yielding 284 observations; studies commonly reported observations from multiple experiments, Cry proteins, or non-target species.

For field studies, we restricted analyses to studies in which the control was a non-*Bt* crop, neither the *Bt* nor the non-*Bt* crop was sprayed with insecticides and abundance of NTOs was measured. This yielded 52 field studies (1514 observations), covering maize (producing Cry1 and Cry3 class proteins), cotton (Cry1 and Cry2), potato (Cry3), rice (Cry1) and eggplant (Cry3).

A weighted mean effect size, Hedges’ *d*, was calculated for each study as the difference between means for *Bt* and control treatments divided by the pooled standard deviation and weighted by sampling variance (Rosenberg *et al*. 2000). Negative values indicate reduced survival or decreased abundance of NTOs in *Bt* compared with control treatments. Random-effect models were used to compare effect sizes between laboratory and field studies. To address the mismatch of organisms tested in laboratory versus field studies, we report analyses for the subset of non-target species that have been sufficiently tested (*n* ≥ 5) in both settings. Analyses were conducted with MetaWin v. 2 (Rosenberg *et al*. 2000). Between-group (laboratory versus field) heterogeneity (*Q*-value) was tested by permutation.

## 3. Results

**Figure 1.**
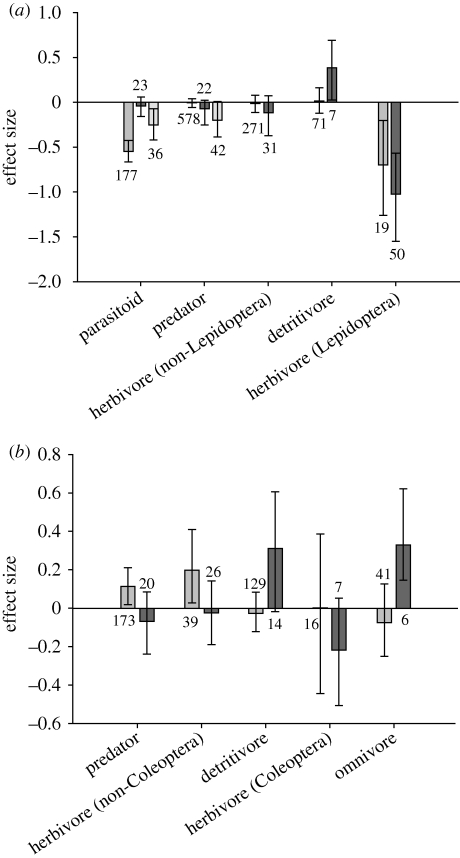
Effect sizes measured in the field versus laboratory for major functional groups of non-target invertebrates. Laboratory studies for parasitoids and predators used either direct or tri-trophic exposure. Positive mean effect sizes (Hedge's *d*) for (*a*) Lepidopteran-active and (*b*) Coleopteran-active *Bt* Cry proteins indicate improved survival or increased abundance when exposed to *Bt* plant tissues or purified Cry proteins relative to a non-*Bt* control. Error bars represent unbiased, bootstrapped 95 per cent confidence intervals. Numbers denote total observations per column. Medium grey bars, field; dark grey bars, laboratory (direct exposure); light grey bars, laboratory (tri-trophic).

For Lepidopteran-active Cry proteins (figure 1*a*), both laboratory and field studies showed expected significant adverse effects on non-target Lepidopteran herbivores, but mean effect sizes did not differ by study type (*Q* = 0.68, *p* = 0.47). Mean effects on non-Lepidopteran herbivores estimated in the laboratory and field studies were statistically indistinguishable from zero and did not differ from one another (*Q* = 0.58, *p* = 0.38). Detritivores were positively affected by exposure to Lepidopteran-active Cry proteins in laboratory studies but not significantly affected in the field, and laboratory and field studies did not differ (*Q* = 1.79, *p* = 0.13).

For parasitoids, direct-exposure laboratory studies failed to detect significant negative effects seen in the field (*Q* = 31.59, *p* = 0.001). By contrast, effects measured in tri-trophic laboratory studies for parasitoids were in the same direction (negative), although significantly smaller in magnitude, compared with effects observed in the field (*Q* = 8.44, *p* = 0.005). For predators, both direct-exposure laboratory studies and studies of field abundance showed non-significant effect sizes that did not differ from one another (*Q* = 0.46, *p* = 0.47), whereas tri-trophic laboratory studies showed adverse effects that were significantly more negative than those measured in field studies (*Q* = 5.33, *p* = 0.030).

For Coleopteran-active Cry proteins (figure 1*b*), laboratory studies involving direct exposure did not reveal statistically significant adverse effects for any functional group examined. These results were consistent with those from field studies for both Coleopteran and non-Coleopteran herbivores. Detritivores and omnivores benefited from exposure to Cry proteins in laboratory but not field studies (detritivores: *Q* = 2.79, *p* = 0.048; omnivores: *Q* = 4.35, *p* = 0.015). Predators were unaffected by *Bt* proteins in laboratory studies, but were more abundant on *Bt* than on control plants in the field (*Q* = 3.96, *p* = 0.05).

**Figure 2.**
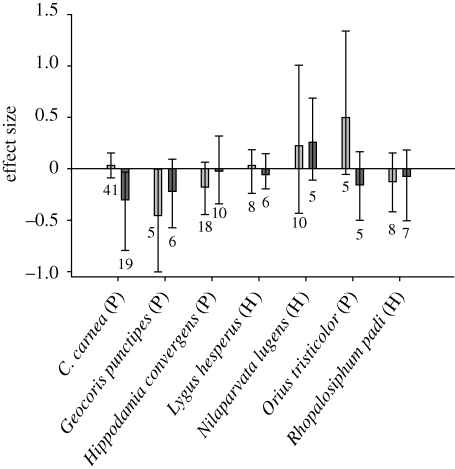
Effect sizes measured in laboratory (direct and tri-trophic studies pooled) versus field studies for species with five or more observations per study type. Data are pooled across Lepidopteran-active and Coleopteran-active Cry protein studies. P, predator; H, herbivore. See figure 1 for description of chart elements. Lighter grey bars, field; darker grey bars, laboratory (tri-trophic and direct exposure).

Analyses for four predator and three herbivore species that have been sufficiently tested in both the laboratory and field support the assumption that laboratory studies accurately predict effects on field abundance (figure 2). However, laboratory studies for *Chrysoperla carnea* yielded negative effects not manifested in the field (*Q* = 3.76, *p* = 0.014).

## 4. Discussion

Laboratory studies of NTOs test for potential hazard, typically under worst-case exposure scenarios, with the assumption that the absence of hazard in the laboratory predicts an absence of ecological harm in the field. To be precautionary, if laboratory studies misestimate effects, they should overestimate risk, thereby triggering follow-up studies. Our meta-analyses indicate that, for the examined functional groups of non-target invertebrates, laboratory studies have, on average, been accurate or conservative in predicting effects of *Bt* crops on field abundance of trophic guilds.

For Lepidopteran-active Cry proteins, laboratory studies correctly predicted the reduced field abundance of non-target Lepidoptera. Positive effects of Lepidopteran-active Cry proteins measured in the laboratory for detritivores were not detected in the field; a likely artefact of protein-deficient control diets used in laboratory studies. Laboratory studies using tri-trophic exposure overestimated ecological effects for predatory insects, representing a successful application of the tiered approach. Recent meta-analyses showed that predator survival is reduced when fed on sublethally damaged caterpillars (low-quality prey) but not on prey unaffected by Cry proteins (Naranjo 2009). Some predators may use prey selection to avoid low-quality prey in the field.

For specialist parasitoids, tri-trophic, compared with direct exposure, studies may better anticipate the effects of *Bt* crops under natural conditions. Adverse effects on parasitoids in tri-trophic exposure studies are largely a function of parasitoids developing within Cry-protein-‘intoxicated’ host caterpillars (Naranjo 2009). Parasitoids reported in field studies with Lepidopteran-resistant *Bt* maize comprised specialist parasitoids such as *Macrocentrus cingulum* Brischke (Hymenoptera: Braconidae). Because this species relies solely on targeted Lepidopteran pests, it is unsurprising that its abundance was reduced in *Bt* maize owing to the effective control of its primary hosts (Wolfenbarger *et al*. 2008). Laboratory studies test for hazard, but field testing may reveal ecological effects that cannot be directly studied in the laboratory. For parasitoids, reduced survival documented in tri-trophic laboratory studies accurately correlates with reduced abundance resulting from host scarcity in the field.

Exposure to Coleopteran-active Cry proteins did not significantly reduce the laboratory survival or field abundance of any functional group examined. Positive effects of Coleopteran-active Cry proteins in the field for non-Coleopteran herbivores and predators may be related to the release of aphids and other sucking insect pests in unsprayed *Bt* potato and the subsequent colonization of predators (Cloutier *et al*. 2008). Positive effects of Cry proteins for omnivores probably arise from the use of protein-deficient control diets in laboratory studies.

Our findings support the validity of the central assumption underlying the tiered approach to risk assessment of transgenic insecticidal crops. Laboratory studies of *Bt* Cry proteins predicted effects that were on average either more conservative than or consistent with effects measured in the field. It would be problematic if laboratory studies predicted no or positive effects, with negative effects revealed only through field studies. One caveat to the support we find for the tiered approach to risk assessment is that laboratory studies should expose NTOs in the full variety of relevant ecological contexts, which may include indirect exposure via an intervening trophic level. Finally, direct toxicity is only one way that *Bt* crops may affect ecosystem services provided by NTOs. Additional research on how *Bt* crops affect the complex interactions within insect communities would benefit our understanding of the long-term implications of widespread adoption of this pest management technology (Lundgren *et al*. in press).
